# The impact of the COVID-19 pandemic on mental health and functional outcomes in Veterans with psychosis or recent homelessness: A 15-month longitudinal study

**DOI:** 10.1371/journal.pone.0273579

**Published:** 2022-08-24

**Authors:** Jonathan K. Wynn, Amanda McCleery, Derek M. Novacek, Eric A. Reavis, Damla Senturk, Catherine A. Sugar, Jack Tsai, Michael F. Green

**Affiliations:** 1 Veterans Affairs Rehabilitation Research & Development Center for Enhancing Community Integration for Homeless Veterans, Veterans Affairs Greater Los Angeles Healthcare System, Los Angeles, California, United States of America; 2 Semel Institute for Neuroscience and Human Behavior, University of California, Los Angeles, Los Angeles, California, United States of America; 3 Department of Psychological and Brain Sciences, University of Iowa, Iowa City, Iowa, United States of America; 4 Department of Biostatistics, University of California, Los Angeles, Los Angeles, California, United States of America; 5 Veterans Affairs National Center on Homelessness Among Veterans, Washington, DC, United States of America; 6 School of Public Health, University of Texas Health Science Center at Houston, Houston, Texas, United States of America; 7 Department of Psychiatry, Yale University School of Medicine, New Haven, Connecticut, United States of America; Universidade Federal do Rio Grande do Sul, BRAZIL

## Abstract

**Background:**

The COVID-19 pandemic has had unprecedented effects on mental health and community functioning. Negative effects related to disruption of individuals’ social connections may have been more severe for those who had tenuous social connections prior to the pandemic. Veterans who have recently experienced homelessness (RHV) or have a psychotic disorder (PSY) are considered particularly vulnerable because many had poor social connections prior to the pandemic.

**Methods:**

We conducted a 15-month longitudinal study between May 2020 –July 2021 assessing clinical (e.g., depression, anxiety) and community (e.g., social functioning, work functioning) outcomes. Eighty-one PSY, 76 RHV, and 74 Veteran controls (CTL) were interviewed over 5 assessment periods. We assessed changes in mental health and community functioning trajectories relative to pre-pandemic retrospective ratings and examined group differences in these trajectories.

**Results:**

All groups had significantly increased symptoms of depression, anxiety, and concerns with contamination at the onset of the pandemic. However, RHV and PSY showed faster returns to their baseline levels compared to CTL, who took nearly 15 months to return to baseline. With regards to functioning, both RHV and PSY, but not CTL, had significant improvements in family and social networks over time. Work functioning worsened over time only in PSY, and independent living increased over time in both RHV and PSY but not CTL.

**Conclusions:**

These results reveal that vulnerable Veterans with access to VA mental health and case management services exhibited lower negative impacts of the COVID-19 pandemic on mental health and community functioning than expected.

## Introduction

The COVID-19 pandemic has affected most people throughout the world, either directly through contracting the SARS-CoV-2 virus or indirectly due to measures intended to slow the spread of the virus [1]. The initial stay-at-home orders and social distancing recommendations enacted by public health authorities in response to COVID-19 dramatically impacted people’s daily social interactions, work, and living situations [2–5]. These impacts are likely to have affected a range of mental health issues and community functioning [5]. The nature of these impacts, though, likely changed over time as the pandemic progressed. Measures intended to slow the spread were lifted, reinstated, and lifted again throughout most of the United States as the pandemic wore on. However, it is not clear if the negative psychosocial impacts of the pandemic after the initial shock of lockdowns and social distancing were short lived or sustained over time.

The pandemic and resulting public health measures have impacted mental health broadly in the general population in the U.S. and across the globe. Several studies have reported increased levels of depression, anxiety, loneliness, and suicidal ideation [6–12]. The pandemic also impacted other areas of mental health, for example with concerns about contamination and obsessive behaviors (such as hand washing) [13]. It has also been well documented that lockdowns and social distancing mandates have negatively affected community functioning, including reductions in number and frequency of social and family contacts, substantial job losses and reductions in pay at the onset of the pandemic, and loss of housing security [14].

Two populations within the U.S. Department of Veterans Affairs (VA) system who may have been particularly vulnerable to the mental health and community functioning impacts of the pandemic are those who have recently experienced homelessness (recently housed Veterans, RHV) or have a psychotic disorder (PSY). Veterans who are receiving services through the VA are an important group to examine because they have resources available, including case management and financial support, that are not uniformly available to other U.S. individuals with these conditions. However, recently housed Veterans and Veterans with PSY commonly lack strong social contacts and therefore might be particularly vulnerable to mental health and social impacts of the pandemic [15]. In a previously published paper from our group [16] that focused on the first several months of the pandemic (through June 2020), we assessed RHV, PSY, and control (CTL; no history of psychosis or chronic homelessness) Veterans. We found increased clinical symptoms (e.g., anxiety, depression, etc.) but relatively little change in social and functional outcomes across groups compared to a retrospective pre-pandemic measure. A recent study in a broad sample of U.S. Veterans showed little change in diagnoses of major depressive disorder or post-traumatic stress disorder due to the pandemic, though middle-aged Veterans showed an increase in generalized anxiety diagnoses [17]. In contrast to the detrimental effects of the pandemic, a national study of U.S. Veterans revealed that a substantial proportion of Veterans reported positive psychological changes and posttraumatic growth during the COVID-19 pandemic [18], which is consistent with pre-pandemic findings of high resilience in the Veteran population [19–21].

It is not clear if these conflicting findings, where some studies find detrimental effects whereas others find some positive effects, are due to the different samples (i.e., vulnerable Veterans vs. a broad Veteran sample) or the timeframe examined. Therefore, in the current study we examined the long-term impact of the COVID-19 pandemic (from the onset of the pandemic through July 2021) on clinical factors (e.g., anxiety, depression) and community integration factors (e.g., social networks, family networks, work) in these two vulnerable Veteran groups, as well as in control Veterans. The current paper extends our previous findings [16] by detailing *changes* in trajectories over a much longer time period (15 months) that was suited for a more sophisticated analytic approach, and to determine if being in a vulnerable group (i.e., RHV or PSY) impacted trajectories more or less than CTL.

## Methods

### Data collection and setting

Data collection occurred between May 2020 –July 2021. All data were collected remotely via telephone interviews from Veteran participants living in Los Angeles County. Data were collected longitudinally over five assessment periods during the pandemic: an initial period (“initial”) and four separate follow-ups (“Follow-Up 1”, “Follow-Up 2”, etc.). Each assessment period lasted approximately two-months. The initial period occurred between May–July 2020; Follow-Up 1 between August–October 2020; Follow-Up 2 between October–November 2020; Follow-Up 3 between January–February 2021; and Follow-Up 4 between April–July 2021. In addition to these assessments, at the initial visit participants were asked to provide ratings on all measures in reference to how they were in January 2020 (i.e., prior to the pandemic, which we refer to as “pre-COVID”). Thus, we relied on participants’ retrospective recall of their clinical and functional status to serve as a pre-COVID measure, which could potentially have caused recall bias due to assessing during the initial phase of the pandemic (i.e., in May 2020). The data from the initial and Follow-Up 1 assessments have previously been published [16], and further details on recruitment and measures assessed are available in that paper. The current paper reports for the first time on data from all four follow-up periods (including the first follow-up data that were previously published) but used a different analytical approach to examine changes over time in our key measures (generalized additive models; see Analytical Approach below). All recruitment and study procedures were approved by the VA Greater Los Angeles Institutional Review Board.

### Recruitment and selection criteria

Selection criteria were intentionally broad for each group and relied on chart diagnoses (or lack thereof) obtained from the VA computerized patient record system (CPRS). All participants in each group consisted of Veterans. For PSY, participants required a psychotic disorder diagnosis (other than substance-induced psychosis). For RHV, participants required a history of chronic homelessness and placement in housing within the past 12 months with a HUD-VASH voucher. Of the RHV, eight received a diagnosis for a psychotic disorder, which was permissible for this group. For CTL, exclusion criteria were no history of a psychotic disorder or evidence of homelessness based on review of CPRS. Thus, controls with other possible psychiatric disorders other than psychosis (e.g., depression, PTSD), substance use disorders, and no history of chronic homelessness, were eligible. Information on diagnosis (including mood disorder, PTSD, substance use disorder, and alcohol use disorder) for each group is provided in S1 Table.

Across the three groups, we identified 956 participants in the Los Angeles area who were potentially eligible and contacted them by phone. Demographic information on the three groups can be found in S1 Table. Following a short description of the study, participants provided informed consent if they agreed to partake in the study. The participant’s contact information was then provided to one of ten clinically trained interviewers who conducted all assessments via phone interview.

### Clinical and functional measures

For clinical factors, we assessed depression (PHQ-9, range 0–27; [22]), anxiety (GAD-7, range 0–21; [23]), obsessive-compulsive thoughts related to germs and contamination (DOCS Category 1: Concerns about Germs and Contamination, range 0–20; [24]), and loneliness (ULS, range 0–60; [25]). For all clinical measures, higher scores indicate worse symptoms. For community functioning, we administered the Role Functioning Scale [26] which assesses four different domains: family networks, social networks, work, and independent living. Scores range from 1–7, with higher scores indicating better functioning.

In addition to the clinical and functional measures, we asked participants at each assessment period how many telephone or video telehealth contacts they made with VA healthcare providers in the prior month and examined the mean (standard error) number of visits across all time points.

### Analytical approach

Our main analytical approach to examine fluctuations over time in the clinical and community functioning factors utilized varying coefficient models, which were implemented using the generalized additive models (GAM) structure via the *mgcv* package version 1.8–34 [27] implemented in R version 4.0.5 [28]. Because the time course of the pandemic has not behaved in a predictable manner (i.e., in a linear or even quadratic fashion), our analytical approach did not assume a rigid parametric form, such as that of a general linear model. Given that we were interested in the differential trajectories between groups in clinical and functional outcomes over time, we analyzed the data using a time varying coefficient model (VCM). With VCM, flexible, smoothed functions for the shape of the trajectories over time can be easily fit and both the linear and non-linear aspects of the data can be modeled without specifying *a priori* what the patterns should look like (e.g., linear, quadratic, etc.).

As we were interested in change in clinical and functional outcomes, we analyzed data in reference to the pre-COVID assessment. That is, we subtracted scores from pre-COVID measures from each respective assessment period to form a change score. While our follow-ups occurred across broad windows, there was wide variability during that window as to when an individual was assessed. To take advantage in the differences in timing, we used specific dates as predictors, rather than follow-up assessment number treated as a factor. We capitalized on the individual differences in the date of the assessment using the VCM approach by calculating how many days passed from the day of a participant’s interview for each assessment relative to the pre-COVID assessment period (for the sake of analyses and figures we set this date to March 1, 2020).

We fit a series of VCMs with each of the clinical or functional factors as the outcome (details of the VCM approach we adopted can be found in S2 File). Within each model, specific contrasts can be extracted from fitting the trajectories enabling us to simultaneously answer two separate questions: 1) does the level of the outcome vary over time within each group; and 2) does the level of the outcome vary differentially over time between groups? We report tests of significance both for time effects related to the pandemic (i.e., within group effects) and whether those effects differed by group (i.e., between group effects). For the statistical results of these analyses, we present the *F*- and *p*-values for the smooth terms for each of the three within group effects, along with the *F*- and *p*-values for the three between group effects, in Table 2, and describe the pattern of findings in the results.

**Fig 1:**
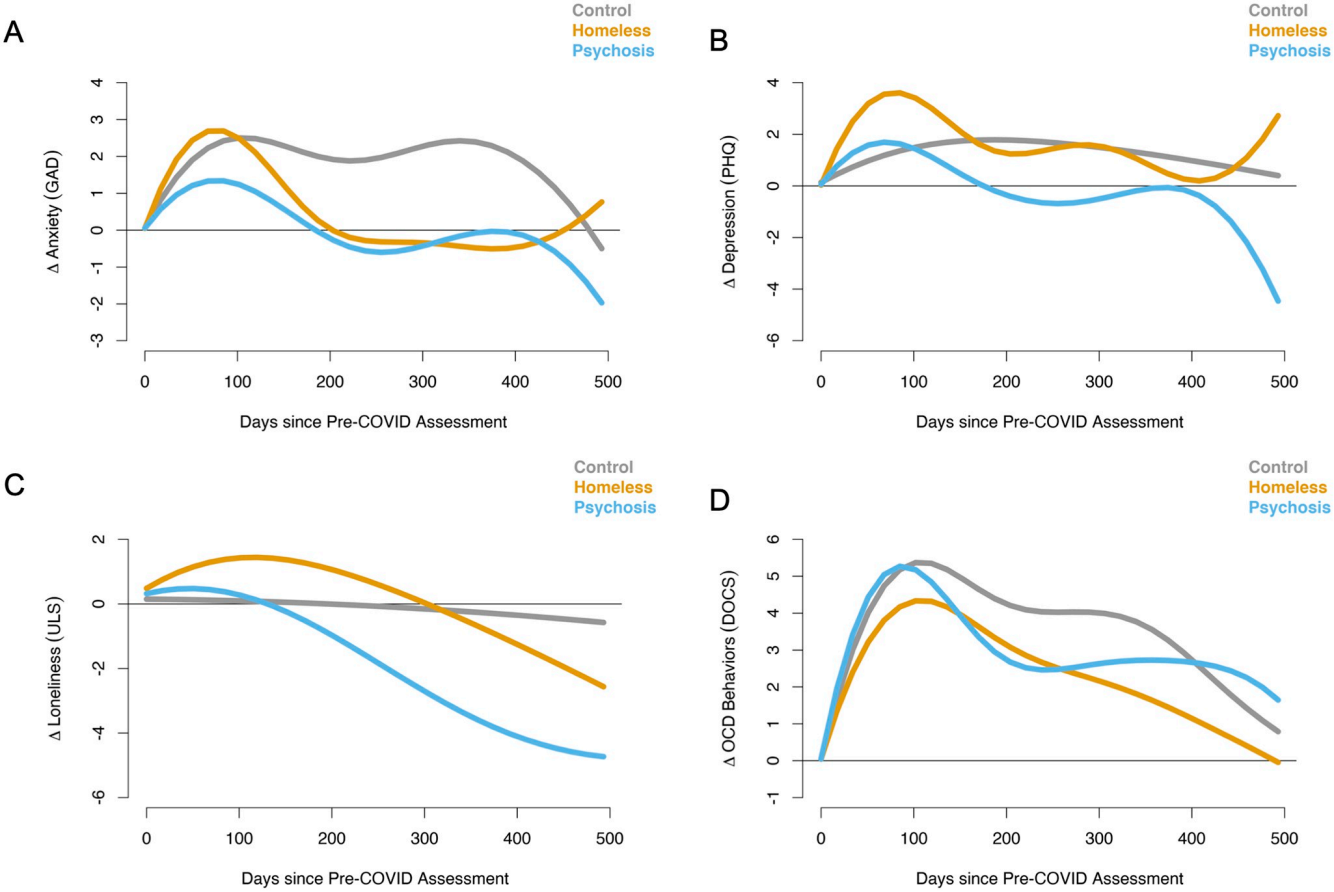
Results for the GAM analyses of clincial outcomes. The smoothed curves represent the change over time in trajectories for controls (CTL; gray), recently housed Veterans (RHV; yellow), and Veterans with psychosis (PSY; blue). In all cases higher scores indicate higher symptoms. The panels show results for A) anxiety, B) depression, C) loneliness, and D) obsessive-compulsive traits.

**Fig 2:**
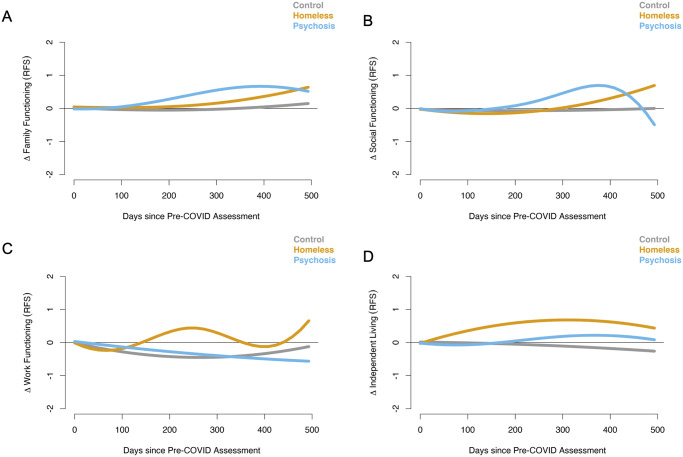
Results for the GAM analyses of community integration outcomes. The smoothed curves represent the change over time in trajectories for controls (CTL; gray), recently housed Veterans (RHV; yellow), and Veterans with psychosis (PSY; blue). In all cases, higher scores indicate better functioning. The panels show results for A) family functioning, B) social functioning, C) work outcomes, and D) independent living.

We present the smoothed curves separately for each group as a function of time for each of the four main clinical outcomes (Fig 1) and the four main community functioning outcomes (Fig 2). We also present the mean difference between pairs of groups (CTL vs. PSY, CTL vs. RHV, and PSY vs. RHV), with 95% confidence intervals, in S1 and S2 Figs. However, the results of formal statistical tests for group differences, which use an omnibus approach, are presented in Table 2. This omnibus approach tests whether the entire time-varying group difference equals zero (hence, the tests are more conservative) and utilize the pointwise confidence intervals as visual guidance on identifying which time periods contributed to significant group differences (seen in S1 and S2 Figs).

## Results

In comparing demographics, controls were significantly older and had more personal education than either the psychosis or homeless groups; however, there were no other group differences (including gender, parental education, ethnicity, or race). Results of a mixed model analysis examining the number of remote (telephone and/or video) telehealth visits revealed a significant main effect of Group, F_2,231.98_ = 5.64, p = 0.004, and Follow-Up Period, F_4,753.12_ = 7.99, p 2.60 x 10^−6^; the Group X Follow-Up Period interaction was not significant, F_8,752.99_ = 0.67, p = 0.714. For Group, there were significantly more visits (estimated marginal means [standard error]) in RHV, 2.54 (0.29), and PSY, 2.49 (0.27), compared to CTL, 1.34 (0.29), p’s < 0.001; there was no significant difference between RHV and PSY. The significant Follow-Up Period main effect was due to decreasing number of visits over the course of the study in each Group.

Table 1 displays the number of participants and percentage of the initial sample (n = 231) for each group at each assessment. We recruited an initial sample of 76 RHV, 81 PSY (42 schizophrenia, 22 schizoaffective disorder, 1 depressive disorder with psychotic features, 9 bipolar disorder with psychotic features, 7 psychotic disorder not otherwise specified), and 74 CTL. At Follow-Up 4, we had an overall retention rate of 68% across groups. We present summarized means and standard error data by follow-up assessment period as bar charts in S3 Fig for clinical measures and in S4 Fig for community functioning measures.

**Table 1:** Sample sizes for each group (and total across groups) at each follow-up period. Percentages in parentheses reflect the percentage of participants from the initial assessment who took part in the respective follow-up.

	*Initial*	*Follow-Up 1*	*Follow-Up 2*	*Follow-Up 3*	*Follow-Up 4*
***Control***	74	66 (89%)	58 (78%)	57 (77%)	50 (68%)
***Homeless***	76	63 (83%)	59 (78%)	55 (72%)	49 (64%)
***Psychosis***	81	74 (91%)	69 (85%)	62 (77%)	59 (73%)
***Total***	231	203 (88%)	176 (76%)	174 (75%)	158 (68%)

### Clinical factors

For anxiety symptoms, all three groups showed an increase in symptoms in the first months of the pandemic (Fig 1A) that then began to return to baseline levels. We found significant time effects for changes in anxiety in all three groups (Table 2). Visual inspection of the smoothed plots suggests that RHV and PSY returned to their baseline levels faster than CTL. The GAM results for the group comparisons confirm this pattern in that CTL showed consistent significantly elevated increases in anxiety compared to RHV and PSY that largely persisted throughout the assessment period (Fig 1A, Table 2). The RHV and PSY showed a return to baseline or decreased symptoms as the pandemic wore on and did not differ from each other.

**Table 2:** Tests of significance and p-values derived from generalized additive models (GAM) analyses, testing for significant changes in trajectories over time for clinical and functional factors (i.e., within group effects) and for significant between-group differences in those trajectories over time (i.e., between group effects).

	Within Group Effects			Between Group Effects		
	Controls (CTL)	Homeless (RHV)	Psychosis (PSY)	CTL vs. RHV	CTL vs. PSY	RHV vs. PSY
**Clinical Factors**	*F value*, *p value*					
Anxiety	**6.83, p < 0.001**	**5.50, p < 0.001**	**3.46, p = 0.005**	**4.81, p < 0.001**	**7.31, p < 0.001**	0.29, p = 0.885
Depression	**4.96, p = 0.004**	**6.33, p < 0.001**	**6.17, p < 0.001**	2.08, p = 0.067	**4.57, p < 0.001**	**3.46, p = 0.004**
Loneliness	0.10, p = 0.910	2.43, p = 0.102	**5.92, p < 0.001**	0.70, p = 0.660	2.31, p = 0.072	1.36, p = 0.250
OCD Behaviors	**27.2, p < 0.001**	**20.3, p < 0.001**	**17.7, p < 0.001**	**3.22, p = 0.022**	**3.54, p = 0.014**	2.47, p = 0.060
**Functional Factors**						
Family	0.54, p = 0.582	**4.63, p = 0.006**	**10.5, p < 0.001**	1.39, p = 0.262	**5.00, p < 0.001**	2.20, p = 0.056
Social	0.07, p = 0.934	**8.53, p < 0.001**	**5.84, p < 0.001**	2.52, p = 0.057	**3.89, p < 0.001**	**3.44, p = 0.002**
Work	2.63, p = 0.073	1.85, p = 0.075	**4.38, p = 0.013**	**2.99, p = 0.006**	0.75, p = 0.525	**3.62, p = 0.013**
Independent Living	2.02, p = 0.133	**23.0, p < 0.001**	**2.79, p = 0.039**	**17.6, p < 0.001**	**3.29, p = 0.011**	**6.91, p < 0.001**

All three groups showed increased depressive symptoms in the first months of the pandemic (Fig 1B). We found significant time effects for changes in depression in all three groups (Table 2). However, PSY had a faster recovery to baseline levels and then, towards the end of the study, improved upon their baseline; both RHV and CTL took longer to return to their baseline levels. The GAM results for the group comparisons confirm this pattern in that PSY showed significant differences compared to both CTL and RHV; the difference in trajectories between RHV and CTL was marginal (i.e., not significant).

Loneliness symptoms did not change significantly over the course of the study in RHV or CTL, but PSY showed significant improvement relative to baseline in (Fig 1C). However, there were no significant between group differences in trajectories over time.

There were substantial increases in OCD-like behaviors in all three groups in the initial months of the pandemic, with all groups showing reductions in these behaviors as the pandemic progressed (Fig 1D, Table 2). Between group comparisons revealed that CTL had significantly higher OCD-like symptoms that persisted longer in comparison to PSY. However, there was no significant difference in symptom trajectories between PSY and RHV.

### Community functioning factors

For family functioning, both RHV and PSY showed significant improvements over time, whereas there was no change in CTL (Fig 2A, Table 2). Group comparisons revealed a significant difference between PSY and CTL, a marginal difference between PSY and RHV, and no difference between RHV and CTL (Fig 2A, Table 2).

The pattern of effects for social functioning was similar to that of family functioning, with both RHV and PSY showing significant improvements in functioning over time, and no change in CTL (Fig 2B, Table 2). There were significant group differences in trajectories between PSY and the other two groups, and a marginal difference between RHV and CTL.

Regarding work functioning, only the PSY group showed a significant change in trajectory, with worsening scores over time; there were no significant changes in trajectories in either RHV or CTL (Fig 2C, Table 2). There were, however, significant group differences between RHV and both PSY and CTL, with RHV showing fluctuating improvements in work functioning whereas the other two groups remained flat or slightly worsened compared to RHV (Fig 2C, Table 2).

Finally, there were significant changes in independent living over time in both RHV and PSY, but no significant change in CTL, where both RHV and PSY showed modest improvements over time (Fig 2D, Table 2). There were significant between-group differences over time amongst all three groups, where RHV > PSY > CTL (Fig 2D, Table 2).

## Discussion

The findings of this longitudinal study on the effects of the pandemic on clinical and community functioning outcomes in vulnerable Veterans showed significant group differences in trajectories for clinical factors as well as for functional factors. All three groups of Veterans showed large negative impacts on mental health outcomes, including depression, anxiety, and OCD-like behaviors, during the first months of the pandemic, relative to retrospective pre-pandemic self-ratings. However, the two vulnerable groups, RHV and PSY, showed faster improvements than CTL on several clinical measures; the CTL group only began to show recovery to pre-COVID levels in the latter part of the study (corresponding to April 2021, more than one year after the onset of the pandemic). Contrary to expectations, none of the Veteran groups showed worsening loneliness, with PSY showing reductions in loneliness over time relative to pre-COVID ratings. Regarding functional outcomes, both RHV and PSY showed improvements in both family and social functioning over time, only PSY showed worse work outcomes over time, and both RHV and PSY showed better independent living outcomes over time. CTL showed no significant changes in any functional outcomes throughout the study. Overall, the results paint a complex picture, showing that vulnerable Veterans were much more resilient to the negative impacts of the pandemic than expected, with the control group showing a more sustained negative impact on clinical outcomes that only began to recover late into the pandemic.

Despite our concerns about the negative mental health impact of the pandemic, the notable lack of greater negative impacts on mental health and community functioning in the vulnerable Veterans in the current study is consistent with a growing literature showing that mental health or community functioning, both in the Veteran [17–21] and general population [29, 30], was not as severely impacted as expected. For example, a large general population cohort in the United Kingdom showed little or no change in mental health during the first six months of the pandemic [29]. A similar pattern across other countries, including the U.S., was seen in healthy older adults who did not show increases in negative mental health outcomes compared to younger populations [30]. Studies in Veterans [17, 18], including our previous paper [16], suggested that Veterans may have good resilience to negative life events which may have impacted the findings. However, we did not examine resilience as a moderating effect in the current paper, and thus cannot explicitly determine if resilience reduced any potential negative impacts on the findings presented herein. While these studies do suggest that the negative impacts of the pandemic on mental health and community functioning were not as large as expected, those studies only covered the early months of the pandemic. Hence, we did not know if this pattern would continue the longer the pandemic wore on. The current study addresses this question directly and shows that the vulnerable groups of Veterans recovered to pre-pandemic levels (and in some cases showed improvements relative to baseline), and that controls (i.e., Veterans without psychosis or a history of chronic homelessness) showed extended elevations in symptomatology that eventually returned to baseline levels at the end of the 15-month study.

There are several potential reasons that might explain why the vulnerable Veterans did not exhibit increased negative impacts on mental health and community integration during the pandemic compared to control Veterans. Chief among these is that many of the vulnerable Veterans had access to and engaged with VA wrap-around services (including mental health and case management services) that were available to them remotely over the course of the study. In the month preceding each assessment, the two vulnerable groups self-reported nearly twice as many telehealth (phone or video) visits with a VA provider. A second possible explanation for our pattern of results is that our Veteran sample was older (mid-50s) and predominantly male (as expected at a VA), which may have been protective as negative mental health outcomes due to the pandemic appear to have been more severe in younger populations and in women, on average [31–33]. Finally, these two vulnerable groups may already be more accustomed to major disruptions, instability, and inconveniences in their lives, so the pandemic may not have seemed especially disruptive, relative to these other experiences.

There are some limitations that should be noted. First, the study relied on retrospective self-reports made by the participants, including the pre-pandemic rating, which could clearly be unreliable or biased based on when we began interviewing participants (May 2020). Second, nearly all participants were male and in the early to mid-fifties, making it impossible to examine if females or younger populations were more or less impacted by the pandemic. Finally, all participants were Veterans, making it difficult to determine if the results would generalize to a non-Veteran population. As mentioned above, the Veteran population has been found to have high resilience, likely due to their military training, past hardships they have overcome, and many medical, psychological, and housing resources provided by the Department of Veterans Affairs that are not readily available to those in the general population. Thus, these unique features of Veterans may differentiate them in many ways from the general non-Veteran population and could have impacted our results.

Despite these limitations, the current findings show that vulnerable Veterans experienced substantial resilience to the negative impacts of the pandemic on mental health and community integration outcomes. These results are consistent with a growing literature showing few negative impacts of the pandemic on mental health and community functioning outcomes in the general community. Our findings also suggest that making mental health counseling or access to community support services widely available in the general community, as they are within the VA, could help to buffer the population against negative mental health and community integration outcomes during future disasters or pandemics.

## Supporting information

S1 Fig Between group differences in changes in clinical trajectories over time. Each column represents one clinical measure, and each row represents one between group comparison (top: controls vs. homeless; mid: controls vs. psychosis; bottom: homeless vs. psychosis). The red line represents the mean difference, and the dashed blue lines and gray shading represent the 95% confidence interval.
(TIF)

S2 Fig Between group differences in changes in functional trajectories over time. Each column represents one functional measure, and each row represents one between group comparison (top: controls vs. homeless; mid: controls vs. psychosis; bottom: homeless vs. psychosis). The red line represents the mean difference, and the dashed blue lines and gray shading represent the 95% confidence interval.
(TIF)

S3 Fig Bar chart of mean values by assessment period for clinical measures. (TIF)

S4 Fig Bar chart of mean values by assessment period for clinical measures. (TIF)

S1 Table Demographic information and clinical diagnoses by group. Demographic information and clinical diagnoses provided separately for each group based on baseline enrollment numbers. Values are either means (standard deviations) or percentages and indicated accordingly.
(DOCX)

S1 File Minimal data set. (CSV)

S2 File Supplemental methods. This file presents specific details for the VCM analyses, as well as supplemental figures.
(DOCX)
