# Supplementary figures and images for: The impact of the COVID-19 pandemic on mental health and functional outcomes in Veterans with psychosis or recent homelessness: A 15-month longitudinal study
Source: PLoS One. 2022 Aug 24;17(8):e0273579. doi: 10.1371/journal.pone.0273579 (PMC9401176; doi:10.1371/journal.pone.0273579)

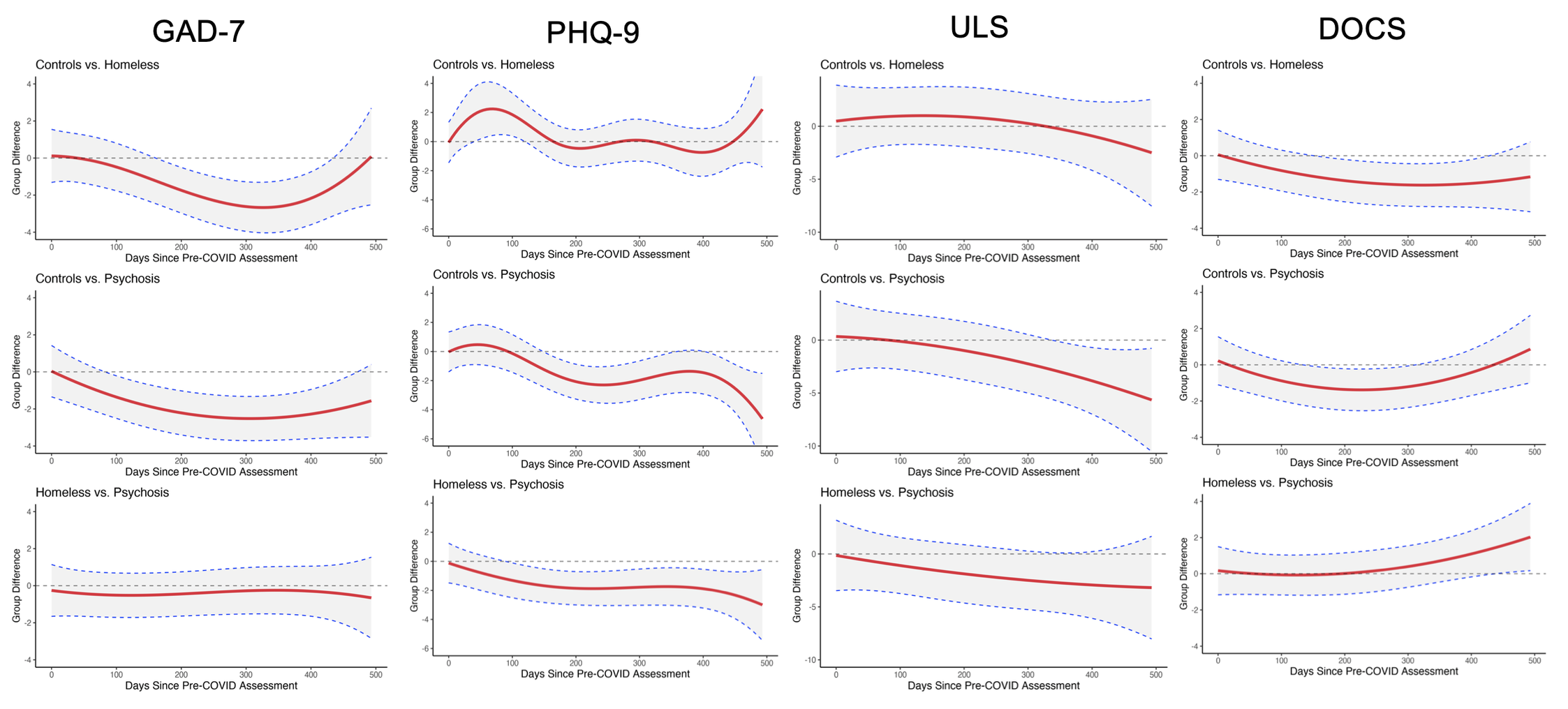

Supplement: S1 Fig — Each column represents one clinical measure, and each row represents one between group comparison (top: controls vs. homeless; mid: controls vs. psychosis; bottom: homeless vs. psychosis). The red line represents the mean difference, and the dashed blue lines and gray shading represent the 95% confidence interval. (TIF) [file pone.0273579.s001.tif]

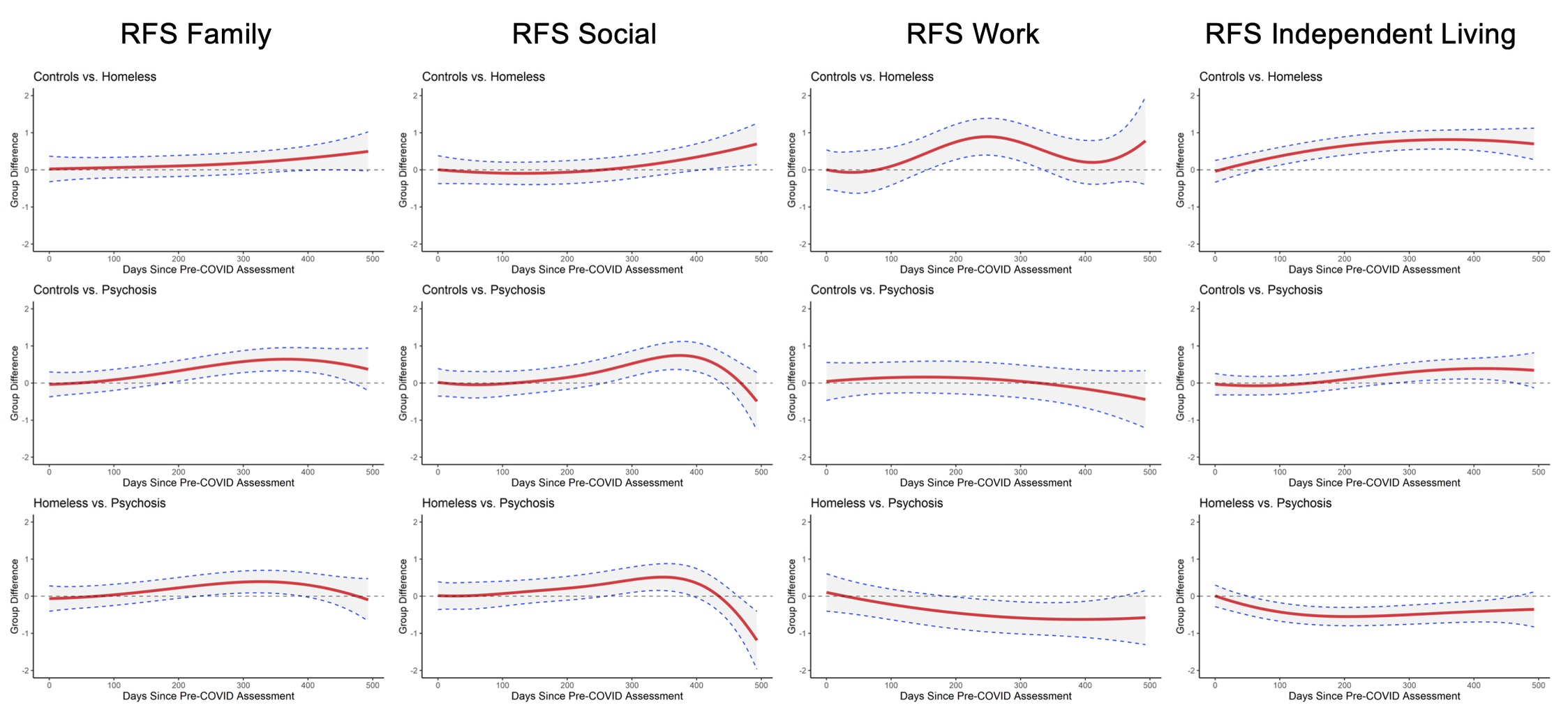

Supplement: S2 Fig — Each column represents one functional measure, and each row represents one between group comparison (top: controls vs. homeless; mid: controls vs. psychosis; bottom: homeless vs. psychosis). The red line represents the mean difference, and the dashed blue lines and gray shading represent the 95% confidence interval. (TIF) [file pone.0273579.s002.tif]

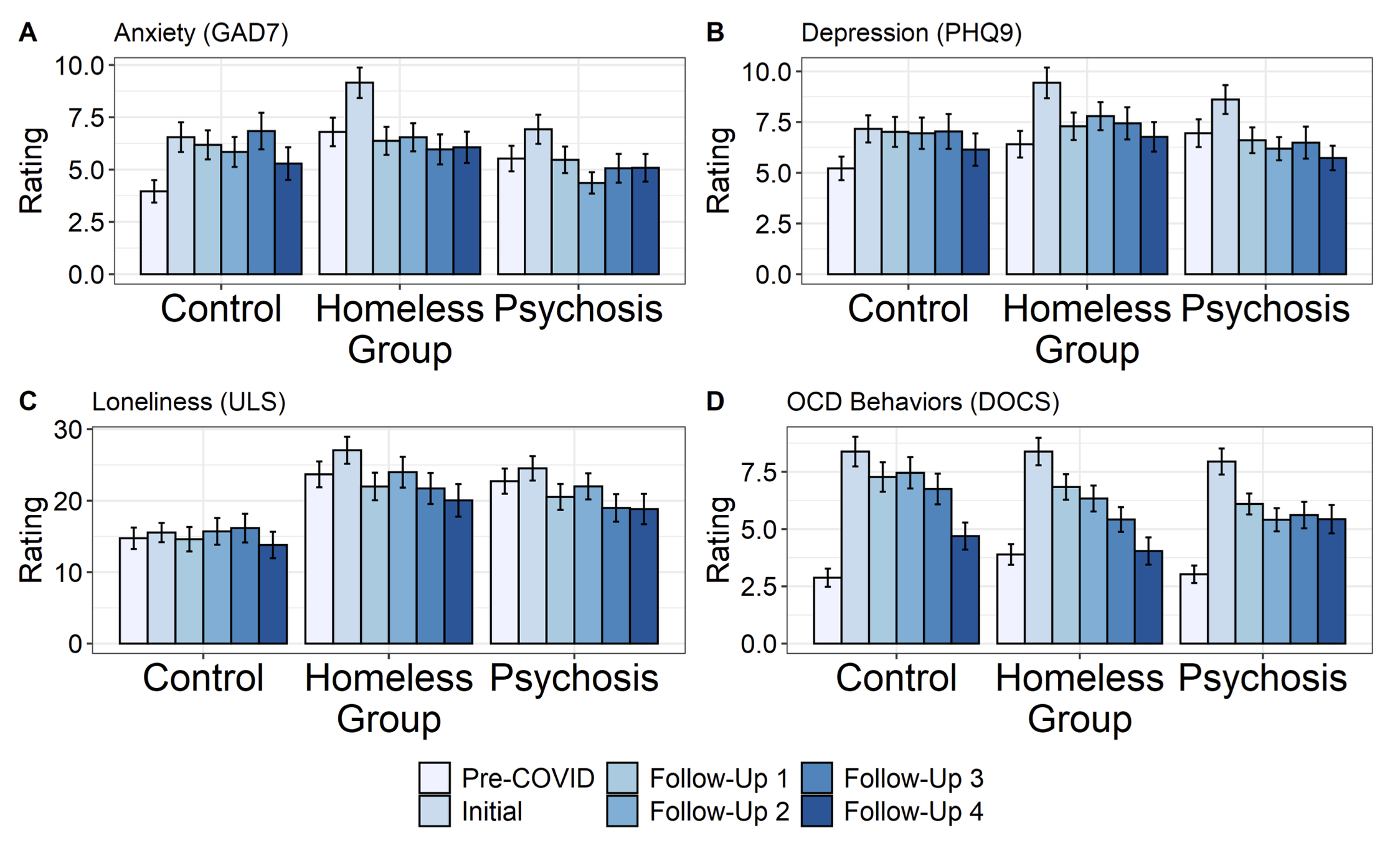

Supplement: S3 Fig — (TIF) [file pone.0273579.s003.tif]

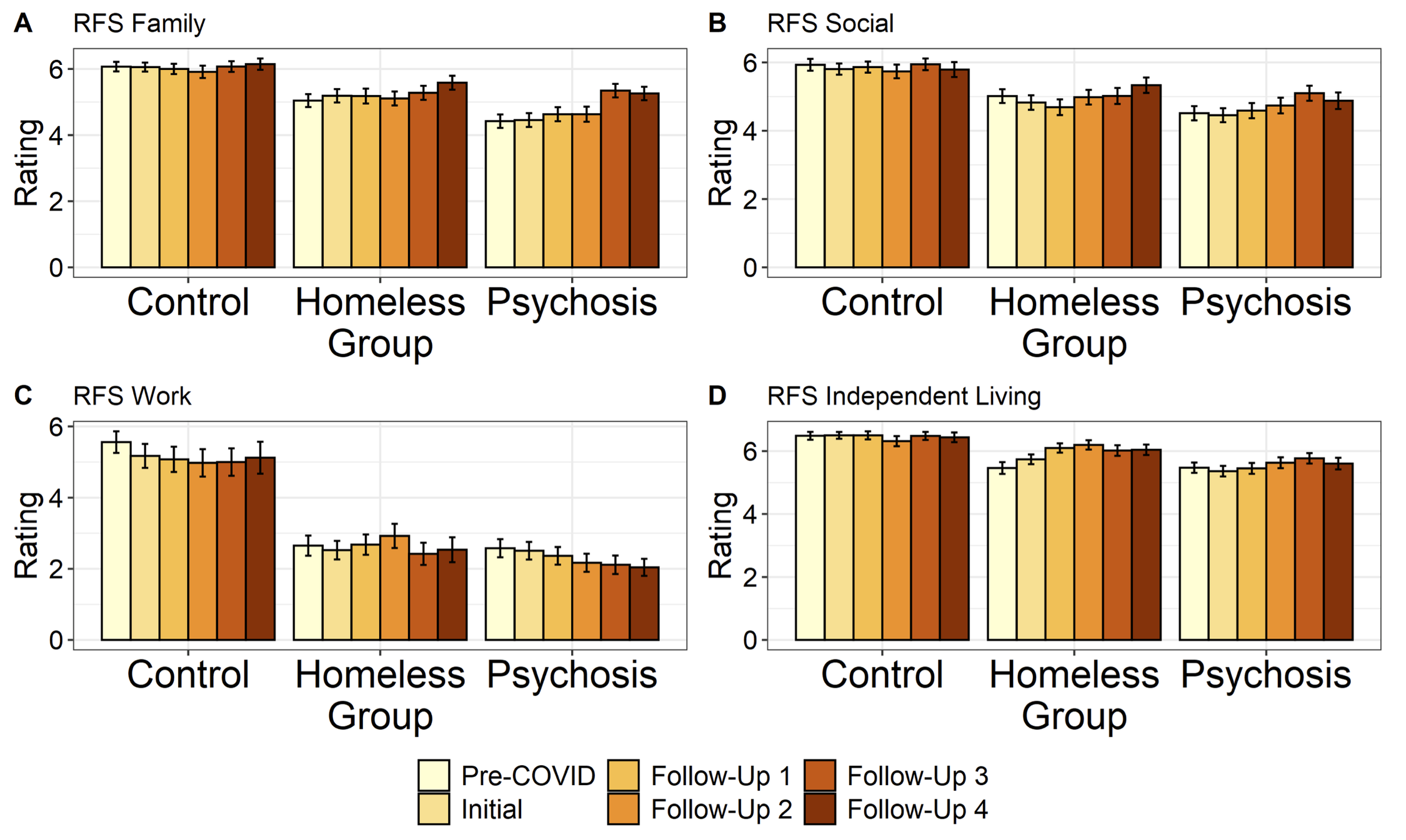

Supplement: S4 Fig — (TIF) [file pone.0273579.s004.tif]
