# Supplementary material for: The impact of the COVID-19 pandemic on mental health and functional outcomes in Veterans with psychosis or recent homelessness: A 15-month longitudinal study
Source: PLoS One. 2022 Aug 24;17(8):e0273579. doi: 10.1371/journal.pone.0273579 (PMC9401176; doi:10.1371/journal.pone.0273579)
Supplement: S1 Table — Demographic information and clinical diagnoses provided separately for each group based on baseline enrollment numbers. Values are either means (standard deviations) or percentages and indicated accordingly. (DOCX) [file pone.0273579.s005.docx]

|  | **Recently Housed (RHV; n = 76)** | **Psychosis**  **(PSY; n = 81)** | **Control**  **(CTL; n = 74)** | **Statistic (*F* or χ^2^)** |
| --- | --- | --- | --- | --- |
| **Demographics** | | | | |
| Gender (F:M) | 10:66 | 9:72 | 11:63 | χ^2^_(2)_ = 0.485, p = 0.785 |
| Age | 51.6 (12.5) | 54.4 (9.8) | 56.5 (9.5) | F_2,228_ = 4.03, **p = 0.019**  CTL > RHV |
| Parental Education (years) | 13.5 (3.1) | 12.9 (3.9) | 13.0 (3.1) | F_2,228_ = 0.74, p = 0.477 |
| Personal Education (years) | 13.4 (1.5) | 13.4 (1.6) | 14.6 (2.0) | F_2,228_ = 12.94, **p < 0.001**  CTL > RHV, PSY |
| Race (B:W:O) | 34:31:9 | 40:29:10 | 28:38:8 | χ^2^_(4)_ = 3.47, p = 0.482 |
| Ethnicity (H:NH) | 21:55 | 16:63 | 19:55 | χ^2^_(2)_ = 1.23, p = 0.541 |
| **Clinical Diagnoses from Medical Records** | | | | |
| Mood Disorder | 60.5% | 23.5% | 47.3% | −−− |
| PTSD | 42.1% | 22.2% | 39.2% | −−− |
| Alcohol Use Disorder | 22.4% | 23.5% | 4.1% | −−− |
| Substance Use Disorder | 38.2% | 33.3% | 9.5% | −−− |

**S1 Table. Demographics and clinical diagnoses for each group.** For age, parental education, and parental education, numbers represent mean (standard deviation).

Note: F = Female, M = Male, H = Hispanic, NH = Non-Hispanic, B = Black, W = White, O = Other, PTSD = posttraumatic stress disorder
