# Supplementary material for: The impact of the COVID-19 pandemic on mental health and functional outcomes in Veterans with psychosis or recent homelessness: A 15-month longitudinal study
Source: PLoS One. 2022 Aug 24;17(8):e0273579. doi: 10.1371/journal.pone.0273579 (PMC9401176; doi:10.1371/journal.pone.0273579)
Supplement: S2 File — This file presents specific details for the VCM analyses, as well as supplemental figures. (DOCX) [file pone.0273579.s007.docx]

Supplemental Methods

Analytical Approach

In the varying coefficient models (VCM), the regression coefficient (beta) is allowed to be a function of time. Our primary predictor for each measure of interest is Group, allowing for individual group effects or differences between them that can change smoothly as a function of time. This allows creating a Group X Time interaction that allows for more complex patterns than simply a difference in slope over time. Note that this means no fixed effects for time are explicitly included in the model, though we did include fixed effects of group to appropriately center the model. Additionally, we allowed the coefficients of group to vary smoothly as a function of time. More specifically the time-varying effects of Group are modeled using penalized B-splines, where the associated smoothing parameters are selected using restricted maximum likelihood (REML). We also included participant-level time-varying random effects to account for correlations between multiple measurements per subject.

The generalized additive model (GAM) analyses was run in R using the *mgcv* package. The general formula used in the *mgcv* pacakge for each of the clinical and functional outcome variables was:

gam(DV ~ Group + s(change_date, bs = "ps", k=30, m = c(2,3), by = Group) + s(PTID,bs="re"), data = temp, method = "REML", drop.intercept=FALSE),

where DV = the dependent variable (e.g., GAD, PHQ, etc.), change_date = the number of days elapsed from the pre-COVID assessment (set to March 1, 2020) for each of the assessments, Group = group membership (homeless, psychosis, control), and PTID = individual subject identification number.

Supplemental Figures

Supplemental Figure 1: The smoothed curves represent between group differences between CTL and RHV, CTL and PSY, and RHV and PSY respectively. The red band indicates the mean group difference, with the dashed blue line and gray shaded area refelecting the confidence interval around that difference. The panels show results for A) anxiety (GAD-7), B) depression (PHQ-9), C) loneliness (ULS), and D) obsessive-compulsive traits (DOCS).

Supplemental Figure 2: The smoothed curves represent between group differences between CTL and RHV, CTL and PSY, and RHV and PSY respectively. The red band indicates the mean group difference, with the dashed blue line and gray shaded area refelecting the confidence interval around that difference. The panels show results for A) family functioning, B) social functioning, C) work outcomes, and D) independent living.

Supplemental Figure 3: Barcharts representing the group means (+/- 1 standard error bar) for each of the clinical outcomes. The panels show results for anxiety (GAD-7), depression (PHQ-9), loneliness (ULS), and obsessive-compulsive behaviors (DOCS). Higher scores indicate worse symptoms.

Supplemental Figure 4: Barcharts representing the group means (+/- 1 standard error bar) for each of the community outcomes. The panels show results from the four domains of the Role Functioning Scale (RFS): family functioning, social functioning, work functioning, and independent living. Higher scores indicate better functioning.
